# The TCR Repertoire Reconstitution in Multiple Sclerosis: Comparing One-Shot and Continuous Immunosuppressive Therapies

**DOI:** 10.3389/fimmu.2020.00559

**Published:** 2020-04-09

**Authors:** Roberta Amoriello, Victor Greiff, Alessandra Aldinucci, Elena Bonechi, Alberto Carnasciali, Benedetta Peruzzi, Anna Maria Repice, Alice Mariottini, Riccardo Saccardi, Benedetta Mazzanti, Luca Massacesi, Clara Ballerini

**Affiliations:** ^1^Dipartimento di Neuroscienze, Psicologia, Area del Farmaco e Salute del Bambino (NEUROFARBA), University of Florence, Florence, Italy; ^2^Department of Immunology, University of Oslo, Oslo, Norway; ^3^Centro Diagnostico di Citofluorimetria e Immunoterapia, Careggi University Hospital, Florence, Italy; ^4^SODc Terapie Cellulari e Medicina Trasfusionale, Careggi University Hospital, Florence, Italy; ^5^Dipartimento di Medicina Sperimentale e Clinica (DMSC), University of Florence, Florence, Italy

**Keywords:** system immunology, T-cell subpopulations, T-cell repertoire diversity, multiple sclerosis, disease-modifying therapies

## Abstract

Natalizumab (NTZ) and autologous hematopoietic stem cell transplantation (AHSCT) are two successful treatments for relapsing-remitting multiple sclerosis (RRMS), an autoimmune T-cell-driven disorder affecting the central nervous system that is characterized by relapses interspersed with periods of complete or partial recovery. Both RRMS treatments have been documented to impact T-cell subpopulations and the T-cell receptor (TCR) repertoire in terms of clone frequency, but, so far, the link between T-cell naive and memory populations, autoimmunity, and treatment outcome has not yet been established hindering insight into the post-treatment TCR landscape of MS patients. To address this important knowledge gap, we tracked peripheral T-cell subpopulations (naïve and memory CD4+ and CD8+) across 15 RRMS patients before and after two years of continuous treatment (NTZ) and a single treatment course (AHSCT) by high-throughput TCRß sequencing. We found that the two MS treatments left treatment-specific multidimensional traces in patient TCRß repertoire dynamics with respect to clonal expansion, clonal diversity and repertoire architecture. Comparing MS TCR sequences with published datasets suggested that the majority of public TCRs belonged to virus-associated sequences. In summary, applying multi-dimensional computational immunology to a TCRß dataset of treated MS patients, we show that qualitative changes of TCRß repertoires encode treatment-specific information that may be relevant for future clinical trials monitoring and personalized MS follow-up, diagnosis and treatment regimes. Natalizumab (NTZ) and autologous hematopoietic stem cell transplantation (AHSCT) are two successful treatments for relapsing–remitting multiple sclerosis (RRMS), an autoimmune T-cell–driven disorder affecting the central nervous system that is characterized by relapses interspersed with periods of complete or partial recovery. Both RRMS treatments have been documented to impact T-cell subpopulations and the T-cell receptor (TCR) repertoire in terms of clone frequency, but, so far, the link between T-cell naive and memory populations, autoimmunity, and treatment outcome has not yet been established hindering insight into the posttreatment TCR landscape of MS patients. To address this important knowledge gap, we tracked peripheral T-cell subpopulations (naive and memory CD4+ and CD8+) across 15 RRMS patients before and after 2 years of continuous treatment (NTZ) and a single treatment course (AHSCT) by high-throughput TCRβ sequencing. We found that the two MS treatments left treatment-specific multidimensional traces in patient TCRβ repertoire dynamics with respect to clonal expansion, clonal diversity, and repertoire architecture. Comparing MS TCR sequences with published datasets suggested that the majority of public TCRs belonged to virus-associated sequences. In summary, applying multidimensional computational immunology to a TCRβ dataset of treated MS patients, we show that qualitative changes of TCRβ repertoires encode treatment-specific information that may be relevant for future clinical trials monitoring and personalized MS follow-up, diagnosis, and treatment regimens.

## Introduction

Multiple sclerosis (MS) is a heterogeneous, organ-specific, inflammatory disease where peripheral, myelin-autoreactive T cells migrate into the central nervous system (CNS) leading to demyelination. It is currently the most widespread autoimmune neurological disease, affecting ~2.5 million people worldwide (1). Approximately 85% of patients are first diagnosed with relapsing–remitting MS (RRMS), and the majority of them are young women (2). Relapsing–remitting MS is characterized by relapses or exacerbations followed by a period of complete or partial remission lasting from a few days to several months.

Over the last 25 years, the immunotherapy of MS and other autoimmune disorders has made considerable progress. Besides first-line treatments that mainly act as immunosuppressants, some of the developed medications are able to rebuild the immune system, inducing profound changes in the lymphocyte repertoire accompanied by long-term therapeutic effects (3).

Two successful RRMS disease-modifying therapies are natalizumab (NTZ), a humanized monoclonal antibody directed against the integrin α4β1 (or Very Late Antigen-4, VLA-4) that prevents the migration of pathogenic T cells into the CNS (4), and AHSCT that resets the immune system and restores immune tolerance by depleting pathogenic adaptive immune cells (5). Both treatments are particularly effective in those patients who have failed previous conventional therapies (6, 7).

Recent reports addressed the potential of these two treatments to impact on T-cell population balance and T-cell receptor (TCR) repertoire. The TCR recognizes peptide antigens in the context of human leukocyte antigen (HLA) molecules and plays a central role in all immune-mediated disorders. High-throughput sequencing has paved the way for investigating TCR repertoire dynamics in peripheral blood mononuclear cells (PBMCs) and tissue-infiltrating lymphocytes. Previously, several studies explored the TCR repertoire in autoimmunity such as diabetes (8, 9) and celiac disease (10), managing to identify an abnormal TCR repertoire shared among patients.

In MS patients, the TCR repertoire was investigated in blood, cerebrospinal fluid, and lesions (11, 12). They found that CD4+ and CD8+ cells are expanded in CNS perivascular spaces and in MS lesions, respectively (13), and that Epstein–Barr virus (EBV)–reactive clones are compartmentalized and expanded in MS brain (14). Recently, Jelcic et al. (4) identified TCRβ sequences shared between an autoproliferative, self-reactive, peripheral CD4+ T cell population and brain-infiltrating cells in one MS patient. Furthermore, de Paula Alves Sousa et al. (15) investigated the peripheral TCR repertoire in MS compared to a viral, neuroinflammatory disease (human T-lymphotropic virus type 1–associated myelopathy/tropical spastic paraparesis), finding a group of TCRβ sequences shared exclusively among MS patients.

Natalizumab, through its mechanism of action, was found to increase the percentage of all immune cell populations in the peripheral blood (PB), especially central memory (CM) T cells, the major fraction of memory CD4+ T cells, and effector memory (EM) T cells (16, 17). In parallel, NTZ increased TCR expansion in PB (18). AHSCT, in turn, was found to result in a complete renewal of CD4+ T cells along with the persistence of selected CD8+ clones (11, 12).

Despite the preliminary progress achieved in understanding how immunomodulatory treatments rebalance the immune system in autoimmune disorders and the involvement of the TCR repertoire, it has remained unknown so far to what extent RRMS treatments impact the TCR repertoire. Insight into treatment-induced TCR repertoire changes is of decisive importance for understanding the effect of RRMS treatments on the molecular level. To quantify the impact of NTZ and AHSCT on patient TCR repertoire, we performed TCRβ sequencing of T-cell subpopulations (naive and memory CD4+ and CD8+) isolated from PB of 15 RRMS patients before and after 24 months of NTZ or AHSCT treatment for a total of 160 sequenced repertoires. We were able to detect treatment-specific molecular and phenotypical traces in RRMS patients, particularly in TCR clonal expansion distribution, clonal persistence across timepoints, TCR repertoire architecture, and serum cytokine (CK) levels.

## Methods

### Patients

All patients included in the study were enrolled at the Clinic of the Second Division of Neurology, Careggi University Hospital, Florence, Italy. Diagnosis of RRMS was performed according to Carroll and McDonald criteria (19). During treatments (AHSCT; NTZ), RRMS patients were followed monthly and evaluated collecting clinical, magnetic resonance imaging (MRI), and laboratory data. Patient characteristics are summarized in Tables 1, 2.

**Table 1:** Clinical characteristics of natalizumab patients.

No. of patients	8
Gender (female:male)	8:0
Age average (min–max)	39.5 (28–45)
Age at treatment initiation average (min–max)	38.5 (21–39)
Type of MS	RRMS
Disease duration average (min–max)	14.3 (8–21)
EDSS T0–T24 average (min–max T0; min–max T24)	3–2.1 (1.5–4.5; 1–3.5)
No. of patients with relapses before natalizumab (AIFA criteria A:B)^#a^	8/8 (5:3)
No. of patients positive for anti-JCV Abs^a^	7/8
No. of patients with relapses^b^	3/8
No. of patients with disease activity (MRI)^#b^	2/8
No. of patients developing anti-natalizumab Absb	1/8
No. of patients with washout relapses	4/8
Therapy^c^	
IFN-β	6/8
Copaxone	1/8
Azathioprine	3/8
No therapies	1/8

^#a^patients who failed the first line therapy, with high disease activity: two relapses in the last year of therapy or one relapse with not complete recovery and up to 9 lesions T2 in MRI or one Gd+ lesions.

^#b^Rapidly evolving patients, with 2 or more relapses in 1 year and 1 or more Gd+ lesions/a significative increment of T2.

^a^At the first natalizumab administration.

^b^During natalizumab therapy.

*^c^Washout period between natalizumab and previous first-line treatments of at least 1 month*.

**Table 2:** Clinical characteristics of AHSCT patients.

No. of patients	7
Gender (female:male)	7:0
Age average (min–max)	39.9 (31–47)
Age at treatment initiation average (min–max)	32.7 (21–47)
Type of MS	RRMS
Disease duration average (min–max)	19.7 (13–38)
EDSS before AHSCT average (min–max)	4.9 (2.5–6.5)
EDSS after AHSCT average (min–max)	4.4 (1.5–6.5)
No. of patients with relapses before AHSCT^a^	6/7
No. of patients with disease progression before AHSCT^a^	0/7
No. of patients with relapses after AHSCT^b^	1/7
No. of patients with disease progression after AHSCT^b^	0/7
Therapy	
Natalizumab^c^	4/7
Cyclophosphamide	3/7
Copaxone	2/7
Azathioprine	2/7
IFN-β	4/7
Mitoxantrone	2/7
No therapies	0/7

^a^During the 2 years before the transplant.

^b^During the 2 years after the transplant.

*^c^Washout period between natalizumab and AHSCT of at least 6 months*.

### AHSCT Patients

Patients were included in AHSCT according to the following characteristics: patient age ranged between 18 and 60 years; Expanded Disability Status Scale (EDSS) between 1 and 6.5; RRMS diagnosis, with MRI activity and/or clinical worsening of the disability despite treatment in the 6 months before the enrollment; and refractory to treatment with NTZ or impossibility to perform this therapy due to allergic reaction to the drugs or presence of neutralizing antibodies. For AHSCT patients who were previously treated with NTZ, the washout period between NTZ and AHSCT was of at least 6 months.

### AHSCT Protocol

Peripheral blood stem cells (PBSCs) from patients were mobilized by cyclophosphamide (4 g/m^2^) and filgrastim (5 μg/kg from). Cells were collected by continuous flow leukapheresis (two whole body blood volumes) when circulating CD34+ cells exceed 20/μL of whole blood; 3–8 × 10^6^ CD34+ cells/kg were collected and stored in liquid nitrogen. Conditioning regimen included carmustine (300 mg/m^2^ per day), cytosine arabinoside (200 mg/m^2^ per day), etoposide (200 mg/m^2^ per day), and melphalan (BEAM) (140 mg/m^2^ per day) for 3 consecutive days. Rabbit anti-T globulin (ATG; Thymoglobuline™) was administered at day +1 and +2 at a total dosage of 7.5 mg/kg. During the reduced intensity conditioning phase, cyclophosphamide (50 mg/kg per day) and ATG (2.5 mg/kg) were administered at +2 and +6 and at +2 and +4, respectively. Biological samples from RRMS patients before (t0) and 24 months (t24) after AHSCT were collected at the Hematology Unit, Careggi University Hospital. Antibiotic and antiviral prophylaxis was administered during the phase of aplasia according to hematology unit practice. At t0, immunosuppressive treatments were stopped at least 1 month before sample collection.

### NTZ Patients

All RRMS patients included in the study failed the first-line treatments and had high disease activity, which means two relapses in the last year of therapy or one relapse with no complete recovery and up to nine lesions T2 in MRI or one gadolinium positive (Gd+), or showed rapidly evolving patients, which means two or more relapses in 1 year and one or more Gd+ lesions or with a significative increment of T2 lesions. Patients included in this study shared the following characteristics: age between 18 and 60 years; EDSS score between 0 and 5.5. Inclusion and exclusion criteria are described in detail in SURPASS study (ClinicalTrials.gov identifier NCT01058005). Presence of anti-JCV and anti-NTZ antibodies in serum was evaluated. Peripheral blood (40 mL) for each MS patient under NTZ treatment was collected at baseline (t0, at least 1 month free of treatment) and after 24 months (t24) of treatment at the Clinic of the Second Division of Neurology, Careggi University Hospital. Natalizumab patients' washout period from previous treatments was of at least 1 month from first-line immunomodulatory treatments [e.g., glatiramer acetate (Copaxone) and interferons].

### Study Approval

This study was performed according to the Declaration of Helsinki. For all AHSCT and NTZ patients, signed informed consent was collected and approved by the Local Ethical Committee (#467/11; #CEAVC12745).

### Clinical Follow-Up of RRMS-Treated Patients

To date, 13 of 15 patients are still followed at the Second Division of Neurology, Careggi University Hospital.

Among the patients who underwent AHSCT, only one (MS032) experienced a relapse at 2 years from AHSCT; of note, MS032 has a family history of MS and interrupted the standard protocol of conditioning chemotherapy to minimize infection risk, subsequently to positivity to the multidrug-resistant strain of *Klebsiella pneumoniae*. MS020, MS029, MS032, and MS034 were treated with NTZ before transplantation, and MS029 is the only one that interrupted NTZ treatment after the development of antibodies against John Cunningham virus (JCV).

Among the NTZ patients, Ty20, Ty21, and Ty25 experienced disease relapses during treatment, whereas MRI brain inflammatory activity (defined as new MRI T2 lesions or Gd-enhancing lesions) was present in patient Ty29. Ty12, Ty17, Ty21, Ty25, and Ty26 suspended the treatment because of JCV positivity. None of the NTZ-treated patients showed a positive signal for anti-NTZ antibodies. Anti-JCV antibodies were present in all patients except for Ty20. During NTZ, Ty26 had an ischemic stroke, and Ty30 developed depression and was lost at the follow-up; no other concomitant disease has been reported. During washout after NTZ, Ty12, Ty20, Ty25, and Ty26 had relapses. Ty21 suspended NTZ for JCV+ and the ineffectiveness of treatment.

Of note, patients from both groups are comparable in terms of demographic (age, sex) and clinical (diagnosis, disease duration) characteristics, and all of them underwent similar treatments before NTZ or AHSCT, with no prevalence of a specific pretreatment among others.

### PBMCs and Serum Collection

Peripheral blood mononuclear cells: whole PB has been collected in heparin-containing tubes; mononuclear cells (MNCs) were collected by density gradient centrifugation using Pancoll (density: 1.077 g/mL; PAN-Biotech, Germany) at 1,500 rpm, room temperature (RT), for 30 min within 6 h from blood collection. Mononuclear cells were cryopreserved in 10% dimethyl sulfoxide in aliquots containing 20 × 10^6^ cells and stored in liquid nitrogen freezer until used.

#### Serum

Serum samples were collected according to the same time schedule and in the same vein puncture as performed for PB collection. For each time point, 10 mL of blood was drawn in serum-separated tubes. Serum was separated from clotted blood by centrifugation at 3,000 rpm for 10 min, RT and then aliquoted and stored in −80°C freezer until used.

### CD3+ Cells Isolation

CD3+ cells were isolated from at least 25 × 10^6^ PBMCs. Peripheral blood mononuclear cells were thawed in defrosting medium (10% fetal calf serum in Dulbecco's phosphate-buffered saline), centrifuged at 1,300 rpm, RT, for 10 min, then counted and used for T-cell isolation by negative immunomagnetic depletion using the Pan T Cell Isolation Kit human (Miltenyi Biotec, Germany), consisting in a Pan Cell Biotin-Antibody Cocktail and a Pan T Cell MicroBead Cocktail that provide the retention of the unwanted cell fraction (CD3-negative cells) and the elution of the CD3+ cells fraction through the LS column placed on a suitable MACS Separator (Miltenyi Biotec). Mean CD3+ cells value per sample was 4 × 10^6^ (±2.2).

### Flow Cytometric Isolation of T-Cell Subpopulations

#### AHSCT Patients

CD3+ cells were washed once in MACS buffer (PBS 1X with 0.5% bovine serum albumin and 2 mM ethylenediaminetetraacetic acid) at 1,500 rpm, RT, for 5 minutes, and then resuspended in RPMI 1640 medium and counted. To identify naive and memory CD4+ and CD8+ T lymphocytes, CD3+ cells were labeled in one tube for 20 min, RT, in the dark, with the following fluorescent antibodies: anti-CD4 APC (clone: OKT4), anti-CD8 PE Cy7 (clone: RPA-T8), and anti-CD45RA FITC (clone: HI100), all from eBioscience, USA. T-cell subpopulations were sorted by FACSAria II flow cytometer (BD Bioscience).

#### NTZ Patients

Purified CD3+ cells were washed and counted as described above. To identify naive, EM, CM CD4+ and CD8+, and TEMRA CD8+ T lymphocytes, cells were divided in two tubes and labeled for 20 min, RT, in the dark, with the following fluorescent antibodies: one tube with anti-CD4 APC (clone: OKT4), anti-CD45RA FITC (clone: HI100), and anti-CCR7 PE (clone: 3D12); and the other one with anti-CD8 PE Cy7 (clone: RPA-T8), anti-CD45RA FITC (clone: HI100), and anti-CCR7 PE (clone: 3D12). All the antibodies are from eBioscience. T-cell subpopulations were sorted by FACSAria II flow cytometer (BD Bioscience).

### HLA II Genotyping

All patients were typed for HLA class II by sequence-specific primer PCR (PCR-SSP) technique using the AllSet+™ Gold SSP Low-Resolution kit (One Lambda).

### Cytokine and Chemokine Evaluation

Serum samples were analyzed for the determination of a panel of 17 CKs and chemokines [interferon γ (IFN-γ), interleukin 4 (IL4), IL1α, IL1β, IL2, IL6, IL8, IL10, IL12p40, IL12p70, IL17, IL23, tumor necrosis factor (TNFα), GM-CSF, CXCL10, CXCL13, MMP9] by Bioplex device (Biorad) using Milliplex assay (Merck Millipore), following the manufacturer's protocol.

### RNA Isolation

RNA was isolated immediately after sorting by using the RNeasy Plus Micro Kit (Qiagen, Netherlands), according to the manufacturer's protocol. RNA purity was assessed by NanoDrop ND-1000 spectrophotometer (EuroClone, Italy) evaluating the 260/280 nm absorbance. RNA concentration (pg/μL), rRNA ratio [28S/18S], and RNA Integrity Number (RIN) were assessed using the RNA 6000 Pico Kit (Agilent, USA), according to the manufacturer's recommendations, and analyzed by Agilent BioAnalyzer 2100.

### TCR Library Generation and RNA Sequencing

The generation of TCR libraries and next-generation sequencing (NGS) of RNA from sorted T cell subsets was performed by the amplification and sequencing services offered by iRepertoire Inc., Huntsville, AL, USA, a company highly specialized in NGS of TCR repertoires. Briefly, TCR Vβ chain libraries were prepared by the patented tem-PCR (for target enriched multiplex PCR technology), developed by iRepertoire Inc., based on the use of nested primers designed to be coamplified in the multiplex assay, and “superprimers,” which act together with the nested primers in two PCR rounds. After purification, amplified TCRβ chain libraries were sequenced by MiSeq Illumina platform, using 250 PER (paired-end read) primers, which cover framework 1 to C-region.

### Statistics

#### Repertoire Data Analysis

Statistical analysis of TCRβ sequence data was performed using the statistical programming environment R (20). Graphs were generated using the R packages ggplot2 (21), ggpubr (22), igraph (23), and ComplexHeatmap (24). Hierarchical clustering of evenness profiles was performed based on correlation-based distance and visualized by heatmaps using the NMF R package (25). Repertoire architecture networks were visualized using Cytoscape (26).

#### Shannon Evenness and Clonal Expansion Profiles

Shannon–Evenness (S-E) and clonal expansion profiles were calculated as previously established by us (27). Briefly, the S-E is defined as the quotient of the exponential of the Shannon entropy and species richness (SR: number of unique CDR3s in a given TCR dataset).

$$\begin{array}{l} \text{Shannon-Entropy}=-\Sigma \;f_{i}\;log\;f_{i}, \end{array}$$

where *f*_*i*_ is the frequency of the *i*th clone in a given TCR dataset.

$$\begin{array}{l} \text{S-E}=exp\left(-\Sigma \;f_{i}\;log\;f_{i}\right)/SR \end{array}$$

Shannon–Evenness is 1 if all clones in a repertoire have the same frequency (an “even” repertoire), or it converges to 0 if very few clones dominate in the repertoire (a “polarized” repertoire), that is, if very few clones have very high frequency and a lot of clones have a very low frequency.

Clonal expansion profiles (also called Evenness profiles) represent Hill diversity profiles scaled by the repertoire SR. Hill diversity profiles are defined as:

Dq=(∑i=1SRfiq)11− q,

where *f*_i_ is the frequency of the ith clone in a given TCR dataset. Clonal expansion profiles are then defined as ^*q*^*D*/SR, where *q* ranges from 0 to 10 with a step size of 0.2. The parameter *q* determines the importance of high-frequency clones in the determination of the *q*-parameterized evenness; the higher *q*, the more high-frequency clones are weighted (28).

### k-mer–Based CDR3 Subsequence Analysis

All CDR3s–amino acid (a.a.) from each patient's TCR repertoire were deconstructed into overlapping subsequences (“k-mers”) of length 3 (*k* = 3) using the tokenizers R package (29). Then, all k-mers were condensed into a k-mer frequency distribution containing each k-mer's frequency across all CDR3s of a given repertoire. Finally, k-mer frequency distributions were correlated across repertoires using Pearson correlation and visualized using hierarchical clustering and heatmap as described above.

### Definition and Quantification of Clonal Persistence (Overlap)

A clone is defined as V-J-CDRβ3 (a.a. sequence). Pairwise clonal persistence between repertoires A and B was calculated as follows:

$$\begin{array}{l} \text{Clonal persistence }\left(\text{A},\text{B}\right)=\frac{A\;\cap \;B}{mean\left(\left|A|,\;|B\right|\right)}, \end{array}$$

where *A* n *B* is the absolute number of persisting clones, and |*A*| signifies the number of clones in repertoire *A*. Clonal persistence was calculated across treatments (t0–t24) for each patient.

### Private and Public Clones

A clone (V-J-CDRβ3 a.a.) was considered private when present in only one repertoire and public when shared at least across two repertoires.

### Network Analysis

Networks of TCRβ repertoires were constructed as previously described (30, 31). Briefly, for each repertoire, a network was drawn using the R package igraph (23) based on the top 10,000 (or less if fewer clones were found in a patient's repertoire) a.a. CDR3 sequences (top 10,000 represented for all repertoires more than 90% of sequencing reads). Within each network, each node is a CDR3, and links between nodes were drawn if the internode Levenshtein distance (LD) is maximally 1 (=1 a.a. sequence change). Degree distributions of each repertoire were also determined using the igraph degree -function. The degree of a node is the number of nodes it has links to (=number of CDR3s 1 a.a. change apart).

### Description of Publicly Available Datasets Used

Our TCRβ data were compared with three other datasets briefly described in the following. The McPAS-TCR database is manually curated and stores TCR sequences associated with various pathologies and antigens based on published literature (32). The McPAS-TCR data here analyzed were downloaded on September 29, 2018. VDJ-DB is a database that stores and aggregates the results of published T-cell specificity assays coupling antigen specificities with TCR sequences (33). The VDJ-DB data here analyzed were downloaded on March 31, 2019.

The Jelcic et al. dataset includes TCR sequences from CD4+ and CD8+ memory cells and brain lesions of 2 MS patients (4). The Jelcic et al. dataset here analyzed was downloaded on November 16, 2018.

### Data Availability of TCRβ Dataset

The TCR sequencing data have been deposited under the following doi: 10.5281/zenodo.3703310.

## Results

### TCRβ Repertoire Sequencing Data Acquisition and Statistics

**Figure 1:**
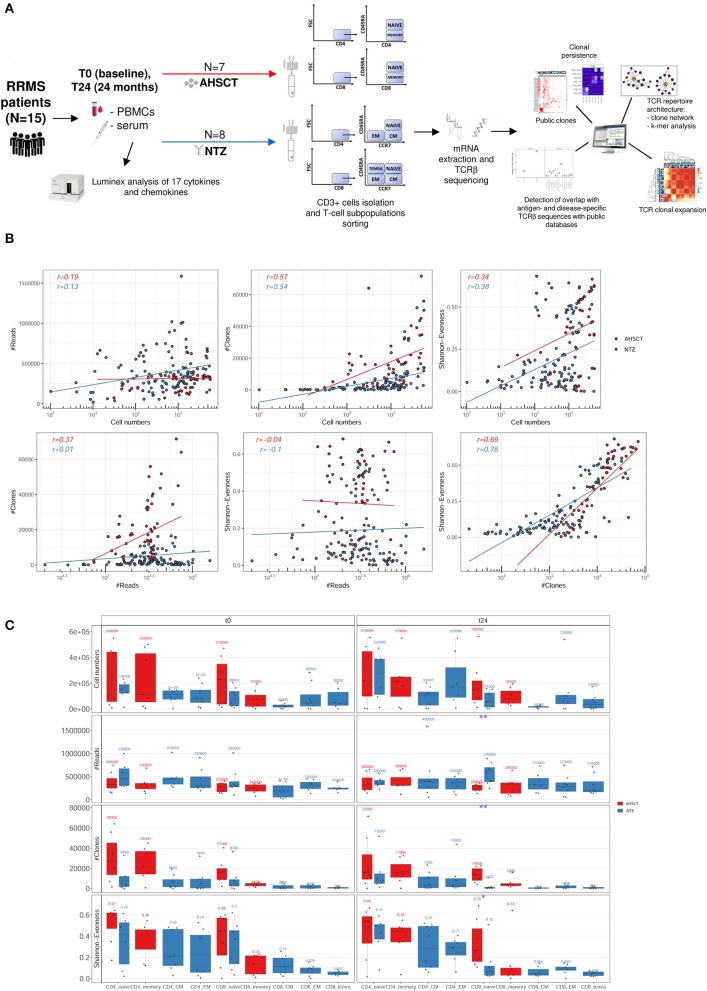
Experimental and computational workflow and T-cell receptor (TCR) repertoire sequencing correlation plots and statistics. **(A)** Peripheral blood mononuclear cells (PBMCs) and serum were collected from relapsing–remitting multiple sclerosis (RRMS) patients (*n* = 15) before (t0) and after 24 months (t24) of AHSCT (*n* = 7) or natalizumab (NTZ; *n* = 8). Serum samples were analyzed for the presence of CKs and chemokines. CD3+ cells were isolated from PBMCs and T-cell subpopulations [naive, effector memory (EM), central memory (CM) CD4+ and CD8+, and terminally differentiated EM (TEMRA) CD8+ from NTZ patients; naive and memory CD4+ and CD8+ from AHSCT patients] were isolated. Subsequently, from total mRNA TCRβ chain (TCRβ) sequencing was performed by iRepertoire Inc. High-dimensional TCRβ data analyses included repertoire sequencing statistics, repertoire architecture, clonal expansion distribution, sequence similarity, clonal persistence across time points, and comparison with public TCRβ databases. **(B)** Plots report the Pearson correlation coefficient (*r*) between reads (#Reads) and cell numbers (left, first row), clones (#Clones) and cell numbers (middle, first row), Shannon–Evenness (S-E) and cell numbers (right, first row), and then between #Clones and #Reads (left, second row), S-E and #Reads (middle, second row), and S-E and #Clones (right, second row) of AHSCT (red points) and NTZ (blue points) repertoires. The S-E quantifies the extent of clonal expansion in each repertoire where values closer to 0 mean high clonal expansion, and values closer to 1 mean low clonal expansion (see section Methods for details). Each point in the correlation plots represents a single TCR repertoire within the two cohorts. Pearson correlation values for each group of patients (in red for AHSCT and in blue for NTZ) are reported on the upper left of each plot. **(C)** Cell number, #Reads, #Clones, and S-E of T-cell subpopulations in NTZ (blue) and AHSCT (red) patients at t0 (left panel) and t24 (right panel). Mean values are shown above each boxplot for all T-cell subpopulations, which are placed on the x-axis for both time points, in this order from left to right: CD4 naive, CD4 memory, CD4 CM, CD4 EM, CD8 naive, CD8 memory, CD8 CM, CD8 EM, CD8 TEMRA. Statistical significance was determined by Wilcoxon test (*p < 0.05; **p < 0.01).

From 15 RRMS patients (eight NTZ and seven AHSCT; see section Methods for patient enrollment and characteristics), we sequenced the TCRβ from T-cell subpopulations [naive, EM, CM CD4+ and CD8+ and terminally differentiated EM (TEMRA) CD8+ for NTZ; naive and memory CD4+ and CD8+ for AHSCT] sorted from PBMCs before (t0) and after 24 months (t24) of treatment, obtaining a total number of 57,330,057 reads and 1,500,769 total number of V-CDR3-J a.a. clones. The experimental workflow is displayed in Figure 1A.

First, to confirm that our sequencing depth allowed sufficient clonal coverage of each repertoire, we determined the correlation of the number of reads, the number of clones (clone definition: V-CDR3-J a.a. sequence), and S-E (see section Methods for a definition of S-E) vs. the number of sorted cells and then between reads and clones number, reads number and S-E, and clones and S-E (Figure 1B). We found the Pearson correlation values to be negative in all cases, thereby signifying sufficient sequencing depth.

We next evaluated the variation, across treatments, of the number of cells, reads (the total number of sequencing reads in for each repertoire), clones (the total number of unique V-CDR3-J a.a. sequences in each repertoire), and S-E by T-cell subpopulation at t0 and t24 (Figure 1C). We found that NTZ, in contrast to AHSCT, induces a significant (*p* < 0.05) repertoire polarization (S-E closer to 0: few clones dominate) in CD8 naive cells from t0 (S-E mean value = 0.3) to t24 (S-E mean value = 0.12), as well as a significant (*p* < 0.01) reduction in the number of clones (t0: ≈8,700, t24: ≈2,000). V gene usage did not differ between RRMS treatments or across timepoints (data not shown).

When following common repertoire statistics on an individual basis, treatment-specific traces are challenging to quantify and visualize (Figure S1). Indeed, when individual repertoire dynamics across timepoints are analyzed, patient variability is high, and no specific trends were apparent. Furthermore, as previously described and shown (33, 34), conventional low-dimensional statistical approaches such as performed in Figure 1 or Figure S1 are not suitable for analyzing the high-dimensional sequence diversity of immune receptor repertoires. Therefore, in the following, we move to more complex measures of immune repertoire comparison. These measures will be applied on an ensemble basis investigating all repertoires at once. As opposed to following patients on a single repertoire basis (Figure S1), ensemble measures allow capturing global trends and minimize potential technological and biological noise inherent to immune receptor repertoire datasets (28, 35, 36) .

### T-Cell Receptor Clonal Expansion Is Differentially Driven by AHSCT and NTZ

**Figure 2:**
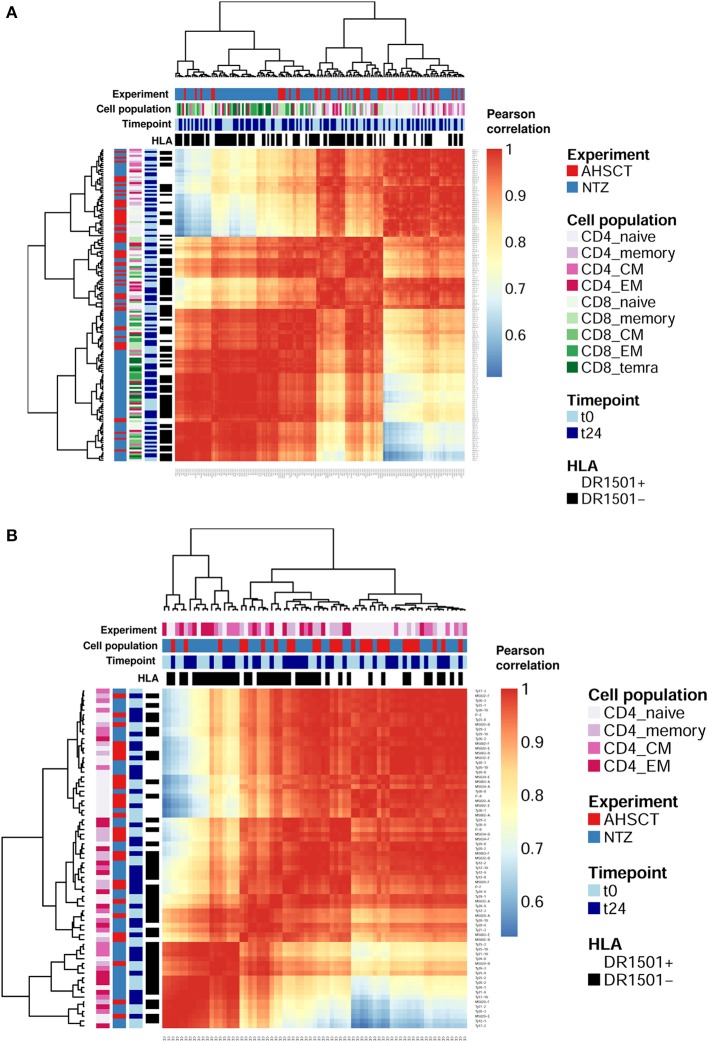
T-cell receptor clonal expansion is differentially driven by treatment. **(A)** Heatmap of the pairwise Pearson correlation between all (CD4 and CD8) Shannon–Evenness profiles (or “state of clonal expansion”). This representation of data allows determining to what extent clustering of clonal expansion is driven by certain parameters such as in our case: treatment, cell subpopulations, timepoint, and HLA class II. Pearson correlation values range from ≈0.5 (blue) to ≈1 (red). Color bars on the top of the heatmap indicate treatment (AHSCT in red, NTZ in blue), T-cell subpopulation (different shades of pink or green are reported for CD4+ or CD8+ subpopulations, respectively), timepoint (t0–t24 in light and dark blue, respectively), and HLA class II of patients when DR1501+ (white) or DR1501– (black). The heatmap x-axis labels indicate T-cell subpopulation, and the y-axis labels indicate sample name. Hierarchical clustering of evenness profiles was performed using correlation-based distance. **(B)** Heatmap of the Pearson correlation between CD4 Shannon–Evenness profiles (or “state of clonal expansion”). Color bars on the top of the heatmap indicate treatment (AHSCT in red, NTZ in blue), CD4+ cell subpopulation (shades of pink), timepoint (t0–t24 in light and dark blue, respectively), and HLA class II of patients when DR1501+ (white) or DR1501– (black). The heatmap x-axis labels indicate T-cell subpopulation, and the y-axis labels indicate sample name.

First, we asked whether clonal expansion is differentially driven by RRMS treatment. To this end, we analyzed clonal expansion using previously established Hill-based evenness profiles (27). Hill evenness profiles have been developed in mathematical ecology and allow a fine-grained comparison of clonal expansion across patient TCRβ repertoires. This analysis evidences differences in clonal expansion among the two treatment groups both for CD4+ and CD8+ T-cell subpopulations (Figures 2A,B and Figure S2A).

Given the documented genetic association of HLA class II haplotype DRB1^*^1501 with MS susceptibility (37), we next asked whether clonal expansion differed by HLA-DRB1^*^1501 type (–/+) and found that HLA class II type impacts on CD4+ clonal expansion (Figure S2A). Although it is known that CD4+ T cells interact with HLA class II, it was interesting to observe a global effect of HLA class II on the entire CD4+ T-cell population encompassing both antigen-specific and non-disease-involved T cells.

To sum up, clonal expansion seems differentially driven by each treatment both in CD4+ and CD8+ subpopulations. Furthermore, CD4+ T cell expansion differed by HLA II type. No difference in clonal expansion was detected between timepoints.

### Clonal Persistence Is Differentially Impacted by RRMS Treatments

We then examined clonal persistence (see section Methods), from t0 to t24, of TCRβ clones across T-cell subpopulations and treatments.

**Figure 3:**
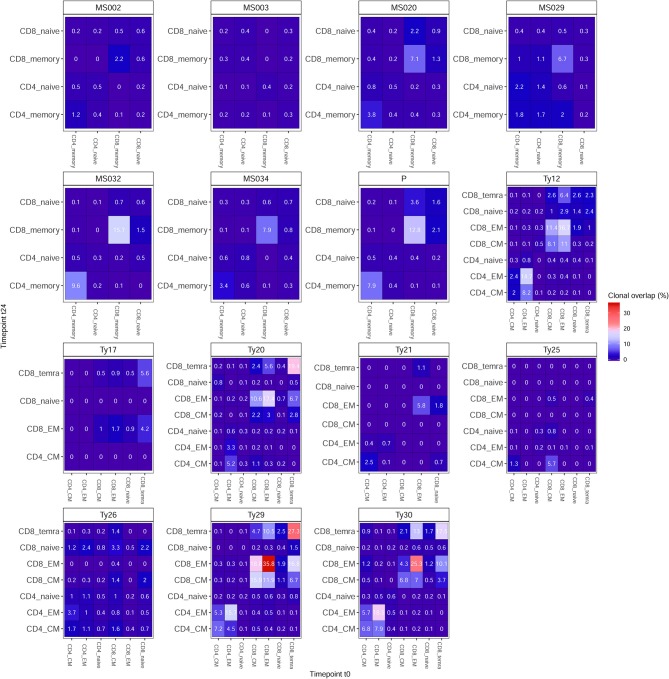
Clonal persistence is differentially impacted by treatment. Percentage of clonal persistence (V-J-CDR3β amino acid overlap) from t0 to t24 is reported for all T-cell subpopulations of AHSCT (patient IDs: MS002, MS003, MS020, MS029, MS032, MS034, and P) and NTZ (patient IDs: Ty12, Ty17, Ty20, Ty21, Ty25, Ty26, Ty29, Ty30) patients. Overlap data are reported across subpopulations per patient as an independent table entitled with the respective patient ID. In all tables, the percentage of clonal persistence is reported for each T-cell subpopulation vs. all other subpopulations tested. See section Methods for details on the numerical determination of clonal overlap.

We found that, for both treatments, clonal persistence is generally higher in the CD4+ and CD8+ memory compartment as opposed to the naive T-cell compartment (Figure 3). In NTZ patients, the percentage of persistent clones across timepoints is higher in CD4+ (0.1–18.5%) and CD8+ (0.4–35.8%) memory subpopulations compared to AHSCT patients (0.2–9.6% and 2.2–15.7% in CD4+ and CD8+ memory cells, respectively). Of note, in both groups of patients, we observed exceptions: patient MS032, who received a different conditioning chemotherapy during transplantation compared to the other patients, showed the highest observed percentage of clonal persistence of all AHSCT patients (15.7% in CD8+ memory and 9.6% in CD4+ memory), whereas in the NTZ group, Ty17, Ty21, and Ty25 (of whom Ty21 and Ty25 experienced a relapse during treatment) differed from the other NTZ patients by showing no or very low clonal persistence in most cell subpopulations.

### AHSCT Patients Share a Higher Number of T-Cell Clones Compared to NTZ

**Figure 4:**
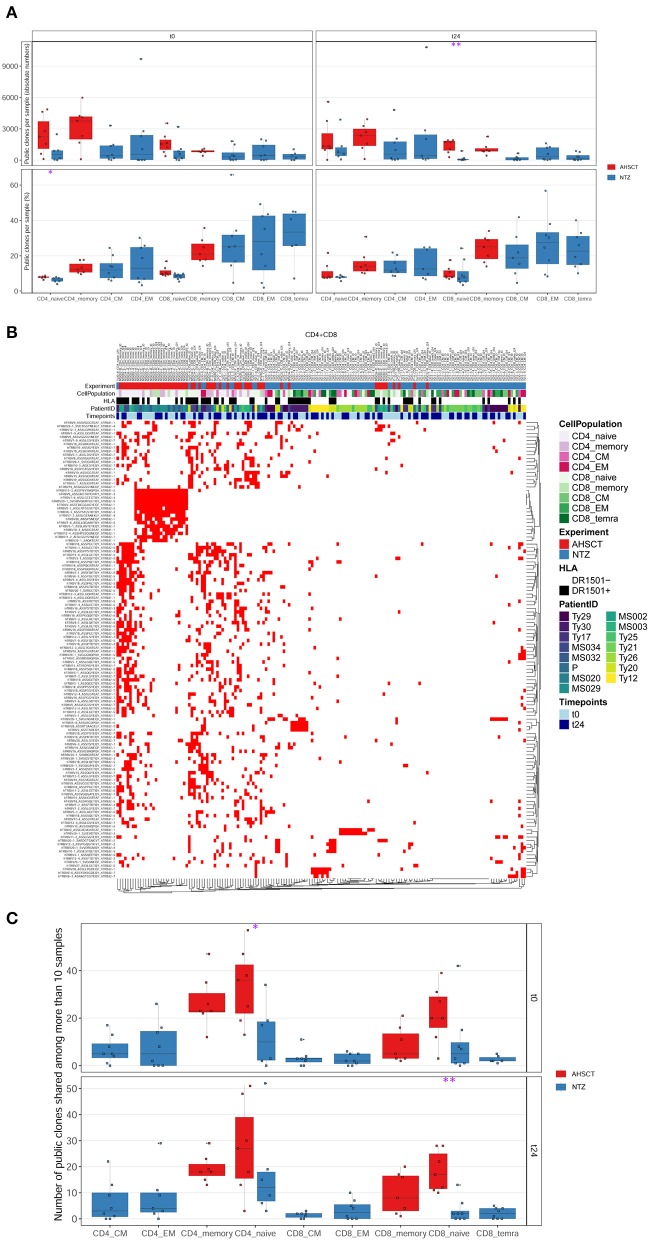
CD8 naive cells of natalizumab patients share less public clones compared to AHSCT at t24, and a group of clones is exclusively shared among AHSCT patients. **(A)** Public clones (V-J-CDR3β amino acid) per sample shown by T-cell subpopulation as absolute number (upper panel) or as a percentage (lower panel) in AHSCT (in red) and NTZ (in blue) patients at t0 (left panel) or t24 (right panel). T-cell subpopulations are reported on the x-axis for both timepoints in this order from left to right: CD4 naive, CD4 memory, CD4 CM, CD4 EM, CD8 naive, CD8 memory, CD8 CM, CD8 EM, CD8 TEMRA. **(B)** TCR clones shared among more than 10 samples visualized by treatment (AHSCT in red, NTZ in blue), T-cell subpopulation (CD4+ or CD8+ subpopulations in different shades of pink or green, respectively), patient ID, timepoint (t0–t24 in light and dark blue, respectively), and by HLA class II of patients when DR1501+ (white) or DR1501– (black). The list of TCR clones is reported on the left and sample name on the top of the heatmaps (it is necessary to magnify the figure to read the labels). **(C)** Statistics of public clones shared among more than 10 samples as shown in **(B)** in the two groups of patients (AHSCT in red, NTZ in blue) shown by T-cell subpopulation at t0 (upper panel) and t24 (lower panel). Statistical significance was determined by Wilcoxon test (*p < 0.05; **p < 0.01).

Next, we investigated the percentage of public clones shared across all patients of both groups, where a clone was defined as public when shared between at least two patient repertoires (Figure 4). When investigating those clones that are highly shared across repertoires, we found a group of clones exclusively shared among AHSCT patients (Figure 4B). These clones were largely not clonally related (different V, J usage, very different CDR3 a.a. sequences) and present in both CD4+ and CD8+ T-cell subpopulations (Figure S3). Quantitatively, the percentage of highly shared public clones was found to be significantly (*p* < 0.05) higher in CD4+ and in CD8+ (*p* < 0.01) naive cells of AHSCT patients compared to NTZ at t0 and at t24, respectively (Figure 4C).

### T-Cell Repertoire Architecture Is Impacted by RRMS Treatment

The architecture of an immune repertoire represents the all-to-all sequence similarity of its composing immune receptor sequences. Thus, repertoire architecture is a direct correlate of antigen recognition breadth: if all immune receptor sequences were very similar to one another, then recognition breadth would be poor, in contrast, if sequences were more dissimilar, a larger space of the antigenome would be covered. Given the importance of repertoire architecture for the function of the adaptive immune response, we asked to what extent patient TCR repertoires differ from patients having undergone different RRMS treatment.

Repertoire architecture (all-to-all sequence similarity within a repertoire) may be quantified in several ways: here we investigated repertoire architecture by both network and subsequence (k-mer) analysis.

**Figure 5:**
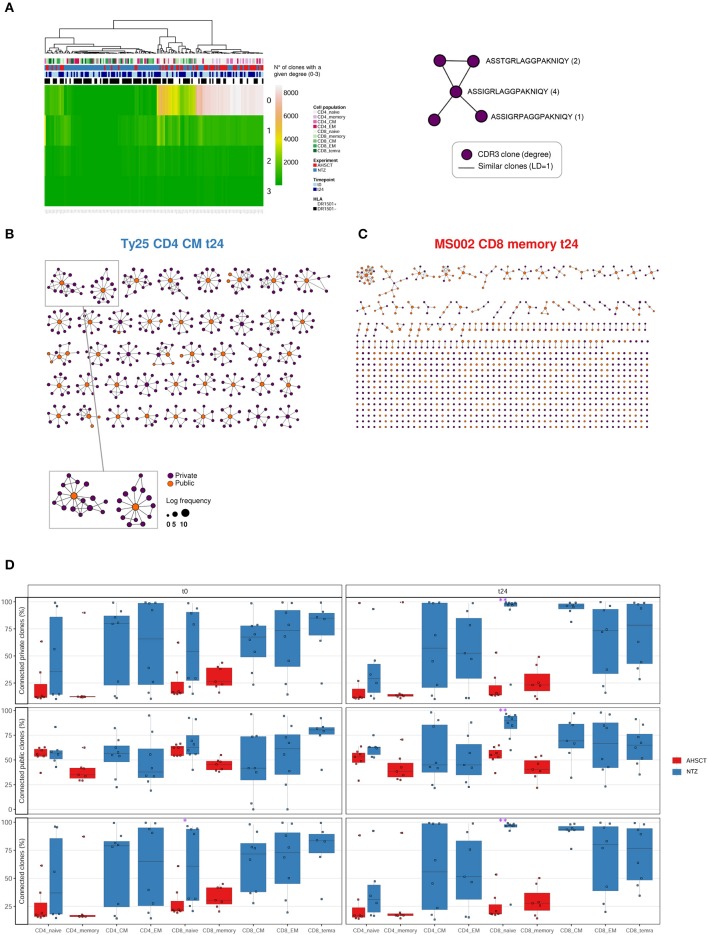
T-cell receptor repertoire architecture is impacted by RRMS treatment. **(A)** Heatmap of the degree distribution of all (private and public) clones. The degree of a clone is the number of clones that is similar to [LD of 1; 1 amino acid (a.a.) change apart]. Color bars on the top of the heatmap indicate treatment (AHSCT in red, NTZ in blue), T-cell subpopulation (different shades of pink or green are reported for CD4+ or CD8+ subpopulations, respectively), timepoint (t0–t24 in light and dark blue, respectively), and HLA class II of patients when DR1501+ (white) or DR1501– (black). The heatmap x-axis labels indicate T-cell subpopulation, and the y-axis labels indicate clone degree from 0 (no clones similar to a clone) to 3 (three clones similar to a given clone). Hierarchical clustering was based on Euclidean distance. **(B,C)** Representative TCR repertoire architectures, displayed as clone networks, of the NTZ patient Ty25, CD4 CM cells, t24 and of the AHSCT patient MS002, CD8 memory cells, t24. Each point, or “node” in the network graph, represents an amino acid CDR3 sequence (CDR3–a.a.) and links between nodes connect CDR3s–a.a. based on LD = 1. Node size scales with log clone frequency and node color indicate private (purple) or public (orange) clones (see legend). **(D)** Percentage of connected private (upper panel), public (middle panel), or all (lower panel) clones in T-cell subpopulations of AHSCT (in red) and NTZ (in blue) patients at t0 (left) and t24 (right). T-cell subpopulations are reported on the x-axis for both time points in the following order from left to right: CD4 naive, CD4 memory, CD4 CM, CD4 EM, CD8 naive, CD8 memory, CD8 CM, CD8 EM, CD8 TEMRA. Statistical significance was determined by Wilcoxon test (^*^p < 0.05; ^**^p < 0.01).

For network analysis of repertoire architecture, we determined the sequence similarity of all sequences composing a repertoire by recording for each CDR3s whether it differed in maximally 1 a.a. from all other CDR3s of repertoire (see section Methods) (30, 31). We focused on changes of 1 a.a. as they may reflect clones with similar antigen-binding profile (38, 39). We found that treatment groups differed in repertoire architecture as evidenced by differences in degree distributions. The degree of a CDR3 in a network is the number of its connections (clones differing by maximally one a.a.). The degree distribution then enumerates the number of CDR3s with an identical degree. AHSCT repertoires have many more clones with degree 0 (no similar clones in the repertoire) compared to NTZ repertoires. This was true for both CD4+ and CD8+ subpopulations (Figure 5A). In CD4+ subpopulations, we observed additional clustering by HLA DRB1^*^1501 (Figure S4). Of interest, differences in clonal connectivity were mostly located in the private part of TCR repertoires. Public clones showed fewer unconnected clones. This is in line with previous studies in healthy human controls (31, 40). Further, the connectivity of public and private clones percentage tended to increase in NTZ compared to AHSCT at t24 as evidenced by a significant (*p* < 0.01) increase in CD8+ naive compartment connectivity from t0 to t24 (Figure 5D).

**Figure 6:**
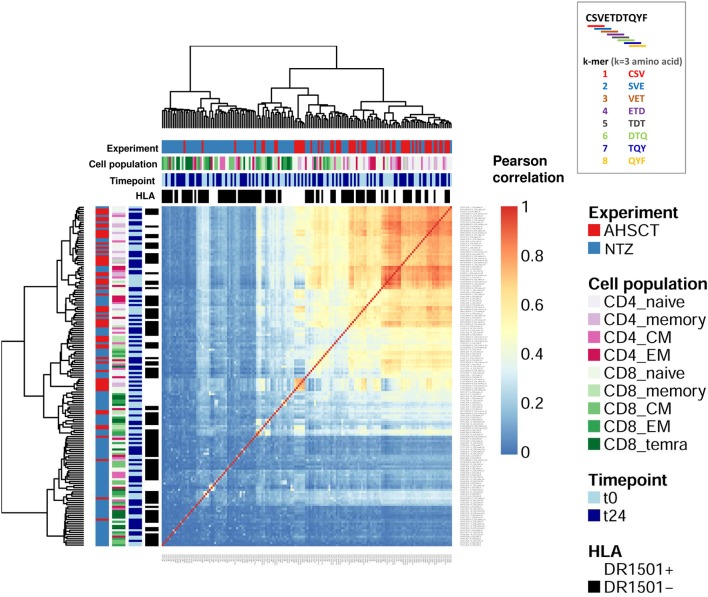
Autologous hematopoietic stem cell transplantation patients show higher TCR sequence similarity compared to NTZ patients. Heatmap of the Pearson correlation of CDR3 k-mer decomposition profiles. Color bars on the top of the heatmap indicate treatment (AHSCT in red, NTZ in blue), T-cell subpopulation (different shades of pink or green are reported for CD4+ or CD8+ subpopulations, respectively), timepoint (t0–t24 in light and dark blue, respectively), and HLA class II of patients when DR1501+ (white) or DR1501– (black). The heatmap x-axis labels indicate T-cell subpopulation, and the y-axis labels indicate sample name. Due to the high number of total samples (160), it is necessary to magnify the figure to read the labels. All CDR3s–a.a. from each patient's TCR repertoire were deconstructed into overlapping sub-272 sequences (“k-mers”) of length 3 (*k* = 3). Subsequently, all k-mers were condensed into a k-mer frequency distribution (k-mer decomposition profile) containing each k-mer's frequency across all CDR3s of a given repertoire. Hierarchical clustering of decomposition profiles was performed based on correlation-based distance.

To complement the network-based repertoire analysis, we additionally investigated the similarity of CDR3 subsequence distributions across repertoires (40). In order to investigate TCR sequence similarity across treatments, timepoints, cell subpopulations, and haplotype, NTZ- and AHSCT-treated patients were analyzed based on subsequence length of 3 a.a. (3-mers). Briefly, all CDR3 sequences of each repertoire were cut into overlapping 3-mers, which were then aggregated to calculate the frequency of each 3-mers. 3-Mer frequency was analyzed across repertoires by correlation. We found that AHSCT patients showed higher TCR subsequence similarity than NTZ patients (Figure 6 and Figures S2B,C) indicating higher preservation of repertoire architecture across AHSCT patients.

To sum up, while NTZ repertoires showed a higher similarity within repertoires (more connected clones), NTZ repertoires exhibited poor preservation of TCR repertoire architecture across repertoires.

### The Majority of Shared Sequences Between RRMS Cohorts and Public Databases With TCRs of Known Disease Association Are Viral Infection Associated

Several viruses, for example, EBV (14), have been suggested as a possible risk factor for MS development. Furthermore, NTZ and AHSCT expose patients to the risk of viral reactivation. Therefore, we asked whether our RRMS dataset included CDR3 sequences already reported in public CDR3 repositories as viral-infection associated.

To this end, we quantified the CDR3 sequence overlap between our dataset and three public CDR3 databases, which store TCR clones of known disease association and/or antigen specificity: McPAS-TCR (32), VDJdb (32), and the TCRβ sequencing dataset from the work of Jelcic et al. (4), who identified a population of proliferative peripheral T cells that share clones with MS lesions (13).

At the time of download (see section Methods), McPAS-TCR (Figure S5A) included 1,297 sequences putatively associated with autoimmune diseases, 6,979 associated with pathogens out of which most are influenza, cytomegalovirus (CMV), EBV, and few MS-associated (118). VDJdb (Figure S5B), including a total of 2,459 human-associated sequences, stored several viral-associated CDR3s, out of which CMV (5,776), human immunodeficiency virus 1 (2,637) and EBV (1,825)–associated CDR3s are the most represented. The Jelcic et al. dataset (Figure S5C) reported information on patient origin (MS1 and MS2), CD4+/CD8+ memory cells, peripheral or brain-infiltrating cells, and auto-proliferative or non-auto-proliferative cells as previously described by authors (4). The highest number of CDR3s (60,449) was identified among the non-proliferative PBMCs of patient MS2.

**Figure 7:**
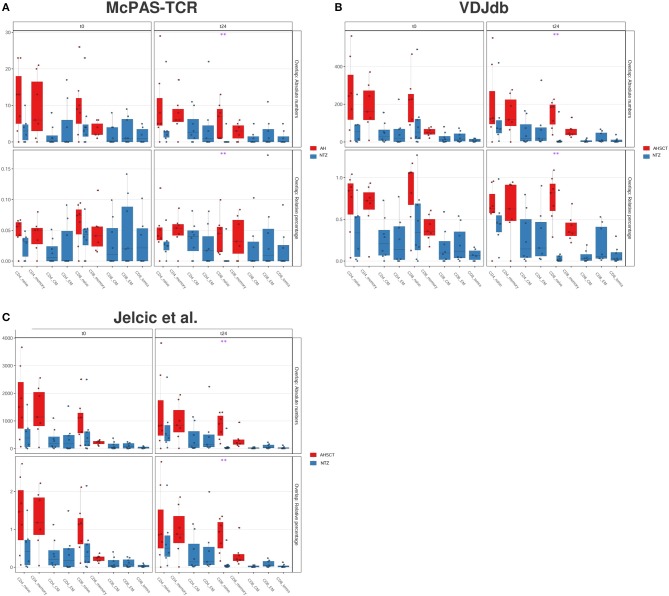
CD8 naive cells of natalizumab patients show a lower CDR3 sequence overlap with public databases compared to AHSCT at t24. CDR3 sequence overlap with McPAS-TCR **(A)**, VDJdb **(B)**, and TCRβ sequencing databases of two MS patients from Jelcic et al. (4) **(C)**. CDR3 sequence overlap is reported as absolute number (upper panel in each graph) or relative percentage (lower panel in each graph) between AHSCT (in red) and NTZ (in blue) patients at t0 (left panel in each graph) and at t24 (right panel in each graph), shown by T-cell subpopulations. Statistical significance was determined by the Wilcoxon test (*p < 0.05; **p < 0.01).

We found that the percentage of overlapping CDR3s between our repertoires and all the examined databases (McPAS-TCR, VDJdb and Jelcic et al., Figures 7A–C) is significantly (*p* < 0.01) lower in CD8+ naive cells of NTZ patients compared to AHSCT at t24.

**Figure 8:**
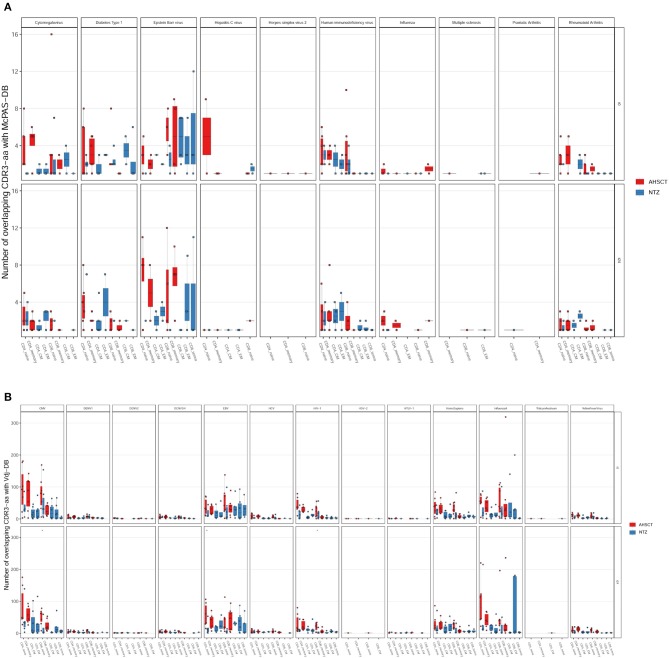
The majority of the shared sequences between RRMS cohorts and public TCR databases are viral infection associated. The absolute number of overlapping CDR3 sequences between McPAS-TCR **(A)** or VDJdb **(B)** TCR databases and AHSCT (in red) or NTZ (in blue) patients at t0 (upper panel in each graph) and at t24 (lower panel in each graph) shown by disease type. Statistical significance was determined by Wilcoxon test (*p < 0.05; **p < 0.01).

Last, comparing our repertoires with McPAS-TCR (Figure 8A) or VDJdb (Figure 8B) displayed by disease type, we found major overlap with viral infection–associated sequences. We found no substantial overlap between our dataset and MS-associated CDR3s (Figure 8A).

### Serum Cytokines Discriminate Between the Inflammatory Status of AHSCT and NTZ

T-cell receptor repertoire variation may be accompanied by alterations in CK production that may govern T-cell differentiation.

Among all the CKs and chemokines measured in serum (see section Methods), we found that TNFα was significantly (*p* < 0.01) lower in NTZ at t0, whereas at t24, it was significantly increased (*p* < 0.05) compared to AHSCT (Figure S6). These findings indicate an increase in TNFα production in NTZ vs. a stable production in AHSCT over the timeline of this study. On the contrary, recall chemokines CXCL10 and CXCL13 are significantly (*p* < 0.05) increased at t24 in AHSCT patients compared to NTZ.

## Discussion

Natalizumab and AHSCT are two effective RRMS treatments, both able to reduce the disease relapse rate in the long term. Hiere, we asked how NTZ and AHSCT impact the adaptive TCR-driven immune response. In the future, such information may facilitate the understanding of the connection between the treatment mechanism of action and patient clinical outcome. In general, we demonstrated that it is possible to detect treatment-specific molecular traces in RRMS patients by high-dimensional analysis of the core molecule of antigen recognition, TCR, in different T-cell subpopulations before and after 24 months of immunotherapy. This is a head-to-head comparison of treatment impact, and the groups at t0, virtually identical, are the control baseline values.

Our results show at t24 a significantly less diverse TCR repertoire of CD8+ naive cells of NTZ patients compared to AHSCT (Figure 1C). CD8+ naive repertoire diversity is commonly higher compared to memory (CM, EM, and TEMRA) CD8+ subpopulations (41), and it represents, along with cross-reactivity, the major strategy adopted by the immune system to face a great variety of antigens (42). Natalizumab has been shown to reduce immune surveillance modulating immune cell populations and subpopulations (43–45). Therefore, the reduced TCR diversity of CD8+ naive cells in our NTZ repertoires may reflect decreased immune protection. This finding is in contrast with previous TCR spectra-typing studies (18) and from PB clone distribution analysis of CD8+ T cells in Rasmussen encephalitis, where the TCR repertoire of CD8+ lymphocytes did not appear significantly impacted after 5 months of NTZ (46). In our NTZ repertoires, the TCR repertoire restriction in CD8+ naive cells may depend on (auto)antigen-specific triggering, the persistence of inflammation or both (47); indeed, serum TNFα is increased from t0 to t24 in NTZ patients and at t24 in comparison to AHSCT (Figure S6). Furthermore, we found that TCR clonal expansion differed by treatment (Figure 2). Specifically, given the role of the HLA-DRB1^*^1501 in MS susceptibility (4, 22, 37), it was interesting to detect the impact of HLA-DRB1^*^1501 on CD4+ clonal expansion (Figure 2B). Major Histocompatibility Complex (MHC) haplotype was previously also shown to be implicated in TCR repertoire biology in other studies (48). We did not observe any clustering based on T-cell subpopulations functional status, for example, EM, CM, and TEMRA in NTZ patients (Figure 2), supporting the fact that pooling memory T cells in AHSCT patients did not affect the comparative analysis between the two groups of patients. Indeed, T-cell subpopulations may be differently distributed in MS, for example, cerebrospinal fluid distribution of memory EM and CM cells, or CD4+ and CD8+ T cell proportion in white matter lesions (49). In our approach, although we specifically addressed TCR repertoire diversity in a longitudinal comparison between different T-cell subpopulations, we did not observe any associations. This may be due to the fact that we were analyzing circulating cells or to the dimension of the sample.

Previously, Muraro et al. (11) described a skewed CD8+ TCR repertoire after AHSCT; in our study, we did not find any similar significant variation when comparing t0 and t24. Furthermore, in the same work, the authors did not detect any clonal persistence among CD4+ subpopulations before and after transplantation, concluding that all clones were depleted by the treatment. Here, we found that in each patient considerable clonal persistence from t0 to t24 is generally present in both groups of patients in CD4/CD8 memory compartment (Figure 3), with some exceptions (MS003, MS032, Ty17, Ty21, and Ty25). Nonetheless, clonal persistence differed by treatment: NTZ preserved CD4+ and CD8+ memory clones to a higher extent compared to AHSCT, where clonal persistence is slightly more pronounced only in memory CD8+ T cells. Of note, patient MS032 showed the highest percentage of clonal persistence from t0 to t24 among all AHSCT patients. Interestingly, this patient interrupted the standard protocol of conditioning chemotherapy for safety reasons and is also the only one who experienced a relapse at t24. In NTZ, where the periphery is supposed to be enriched by pathogenic clones due to the NTZ's mechanism of action, clonal persistence is generally higher compared to AHSCT patients, except for Ty17, Ty21, and Ty25, whom two-thirds (Ty21 and Ty25) had a relapse during treatment. In light of these results, it is tempting to speculate that evaluating clonal persistence in specific memory T-cell subpopulations may contribute to predict the patient outcome to treatment in the future.

The repertoire architecture describes the TCR repertoire organization by quantifying clone-to-clone sequence similarity. In this context, T-cell clones carrying similar CDR3s–a.a. may have been selected by a common force, such as V(D)J recombination, thymic selection, antigen encounter, and, in this case, immunomodulatory treatment (30, 40, 50). We found that methods quantifying repertoire architecture, network, and k-mer subsequence similarity analysis were able to readily distinguish between treatments (Figures 5, 6). We found that both CD4 and CD8 architectures differed across treatment groups, and that effect was observable both in the public and private compartment. More specifically, we found a greater percentage of connected private and public clones at t24 in CD8+ naive T cells of NTZ group compared to AHSCT (Figure 5D), together with a lower number of public clones (Figures 4A,C). This observation in CD8 naive cells of NTZ patients, along with a significantly lower number of shared CDR3s with published databases (Figure 7) and the decreased number of clones and S-E mean value at t24 (Figure 1C), may be linked to a mild impact of NTZ on VLA-4 expression on the surface of naive cells compared to memory subpopulations, as previously documented (51), with the consequence of an increased thymic retention of this cell population (52). It would be worthwhile to investigate how long this effect lasts after NTZ interruption and if it may be, in part, the culprit of the higher incidence of infection-related adverse events observed in patients that shifted treatment, for example, from NTZ to AHSCT (53). Furthermore, the higher TCR sequence similarity in AHSCT patients compared to NTZ (Figure 6) may depend on the fact that AHSCT patients underwent the same posttransplant protocol, remaining at least 1 month in a sterile chamber, sharing the same nutritional restrictions and the same antimicrobial therapies. Our data do not suggest a role of previous first-line therapies in the variation of TCR dynamics; therapies were randomly distributed in the two cohorts and are variable intra and inter patients groups.

The fact that treatment groups may be differentiated based on public clones as well as the fact that we found a group of clones that were uniquely observed in the AHSCT compartment let us think that, in the future, it would be worthwhile to perform a study on a larger cohort with more patients and deeper sequencing depth, possibly also in other T-cell subpopulations such as regulatory T cells (36). Furthermore, it is of interest that repertoires of both groups of patients were enriched in viral disease–associated TCRs (Figures 8A,B). Whether these TCRs are involved in therapy outcome remains to be determined. Recently, our group analyzed antibody titers in patients after transplantation (data presented at EBMT 2018, *Bone Marrow Transplantation* 2018; 53:210) and found that, after engraftment (24 months), the range of a loss of protection is between 11% and 44%. Indeed, in a subgroup of patients, the immune memory against viruses was found to be preserved; when it is not, revaccination is recommended after careful evaluation of the possible disease reactivation risk (54). Indeed, the interplay between MS and viral response in terms of pathogenesis has always been suggested, and it is intriguing that in our samples, among virus-associated sequences, EBV-associated ones are the most represented (Figure 8). On the other hand, there are still few MS-associated sequences in the public TCR datasets, probably because they are difficult to be defined in the absence of a known MS antigen, and this may be in part responsible for our results. In general, more information is needed on how distributions of antigen-specific TCRs vary across disease states (35, 36).

To sum up, repertoire sequencing statistics examined longitudinally patient by patient suggest that, even though we were able to distinguish the two treatments, describing the TCR repertoire dynamics is a personal matter: each patient has his/her own repertoire and shows an individual treatment response (Figure S1). However, when leveraging computationally immunology investigating multiple different aspects of repertoire diversity, we found that clonal expansion (Figure 2), clonal persistence (Figure 3), public clones number (Figure 4), and repertoire architecture (Figure 5) differentiate readily between treatment groups. Specifically, for NTZ, there is a trend toward a status of incomplete immune reconstitution that becomes significant in CD8+ naive T cells, whereas in AHSCT there is an almost complete TCR repertoire reconstitution in all subpopulations investigated.

In conclusion, our work demonstrates that investigating MS patient TCR repertoires during immunomodulatory treatments by deep sequencing methodologies applied on T-cell subpopulations and followed by rigorous statistical analysis enables to distinguish the different treatment effects on immune response and suggests a possible bridge between molecular data and clinical data. In light of this, we believe that, in the future, tracking TCR repertoire dynamics during RRMS treatments might accompany the clinical evaluation of patients as a supportive tool to understand the disease pathogenesis, the disease-modifying treatment impact on adaptive immune response, and treatment outcome and risk.

## Data Availability Statement

The TCR sequencing data has been deposited under the following doi: 10.5281/zenodo.3703310. Other raw data supporting the conclusions of this article will be made available by the authors, without undue reservation, to any qualified researcher.

## Ethics Statement

The studies involving human participants were reviewed and approved by Careggi University Hospital, Florence, Italy, Local Ethical Committee (#467/11; #CEAVC12745). The patients/participants provided their written informed consent to participate in this study.

## Author Contributions

RA conducted the experiments and contributed to writing the manuscript. VG analyzed data and contributed to writing the manuscript. AA and EB conducted the experiments. BP performed cell sorting experiments. AC conducted Luminex experiments. AR acquired clinical data. AM acquired clinical data and contributed to discussion. RS acquired clinical data. BM performed the experiments. LM acquired and analyzed clinical data. CB designed the research and wrote the manuscript.

### Conflict of Interest

The authors declare that the research was conducted in the absence of any commercial or financial relationships that could be construed as a potential conflict of interest.
