# Supplementary material for: The TCR Repertoire Reconstitution in Multiple Sclerosis: Comparing One-Shot and Continuous Immunosuppressive Therapies
Source: Front Immunol. 2020 Apr 9;11:559. doi: 10.3389/fimmu.2020.00559 (PMC7160336; doi:10.3389/fimmu.2020.00559)
Supplement: Supplementary file 1 [file Data_Sheet_1.PDF]

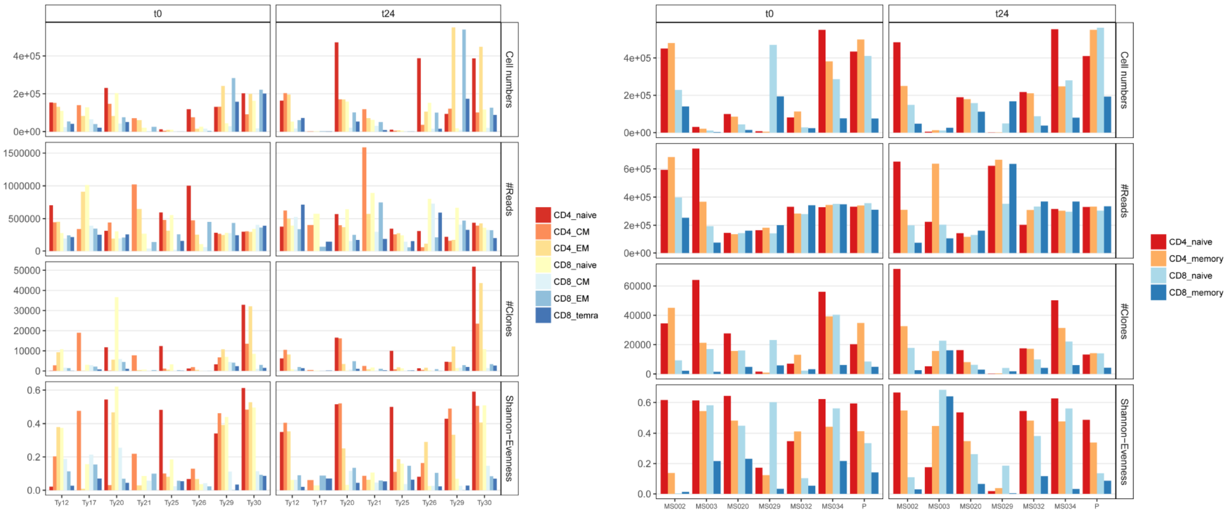

Figure S1. Individual TCR repertoire dynamics are characterized by high variability and no specific trends. Cell numbers, number of reads (#Reads), number of clones (#Clones) and Shannon-Evenness (S-E) reported patient by patient for T-cell subpopulations of NTZ (left) and AHSCT (right) patients at t0 (left panel in each graph) and t24 (right panel in each graph). Each bar represents a T-cell subpopulation.

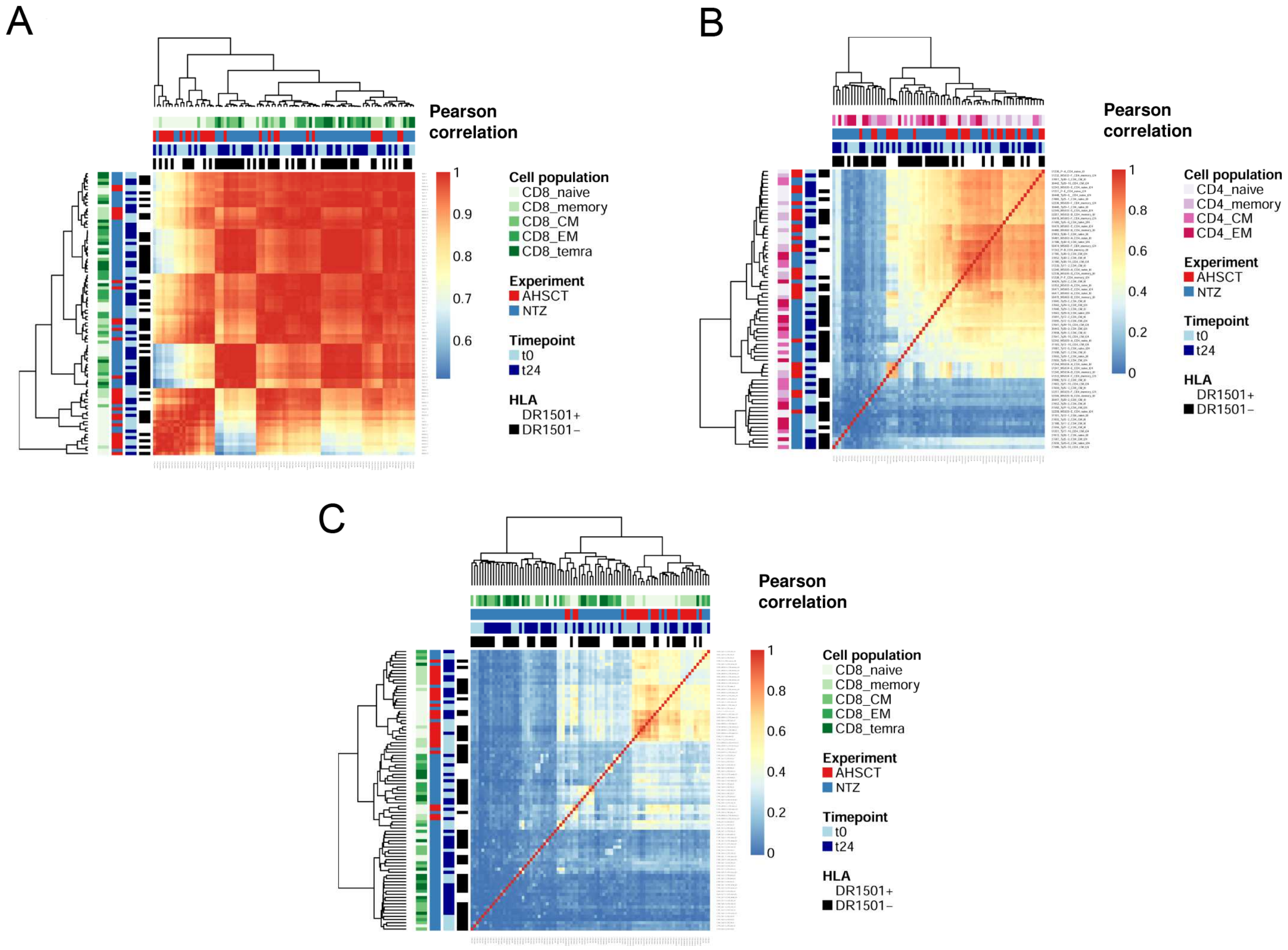

Figure S2. HLA class II type impacts on TCR clonal expansion of CD4+ cell subpopulations and AHSCT patients show higher TCR sequence similarity compared to natalizumab patients. (A) Heatmap of the Pearson correlation of all Shannon-Evenness values (or "state of clonal expansion"). Color bars indicate treatment (AHSCT in red, NTZ in blue), CD8+ T-cell subpopulation (shades of green), timepoint (t0-t24 in light and dark blue, respectively) and HLA class II of patients when DR1501+ (white) or DR1501- (black). The heatmap x-axis labels indicate T-cell subpopulation and the y-axis labels indicate sample name. Hierarchical clustering of evenness profiles was performed using correlation-based distance. **(B, C)** Heatmaps of the Pearson correlation of CDR3 k-mer decomposition profiles. Color bars indicate treatment (AHSCT in red, NTZ in blue), CD4+ (B) or CD8+ (C) T-cell subpopulation, timepoint (t0-t24 in light and dark blue, respectively) and HLA class II of patients when DR1501+ (white) or DR1501- (black). The heatmap x-axis labels indicate T-cell subpopulation and the y-axis labels indicate sample name. Hierarchical clustering of evenness profiles was performed using correlation-based distance.

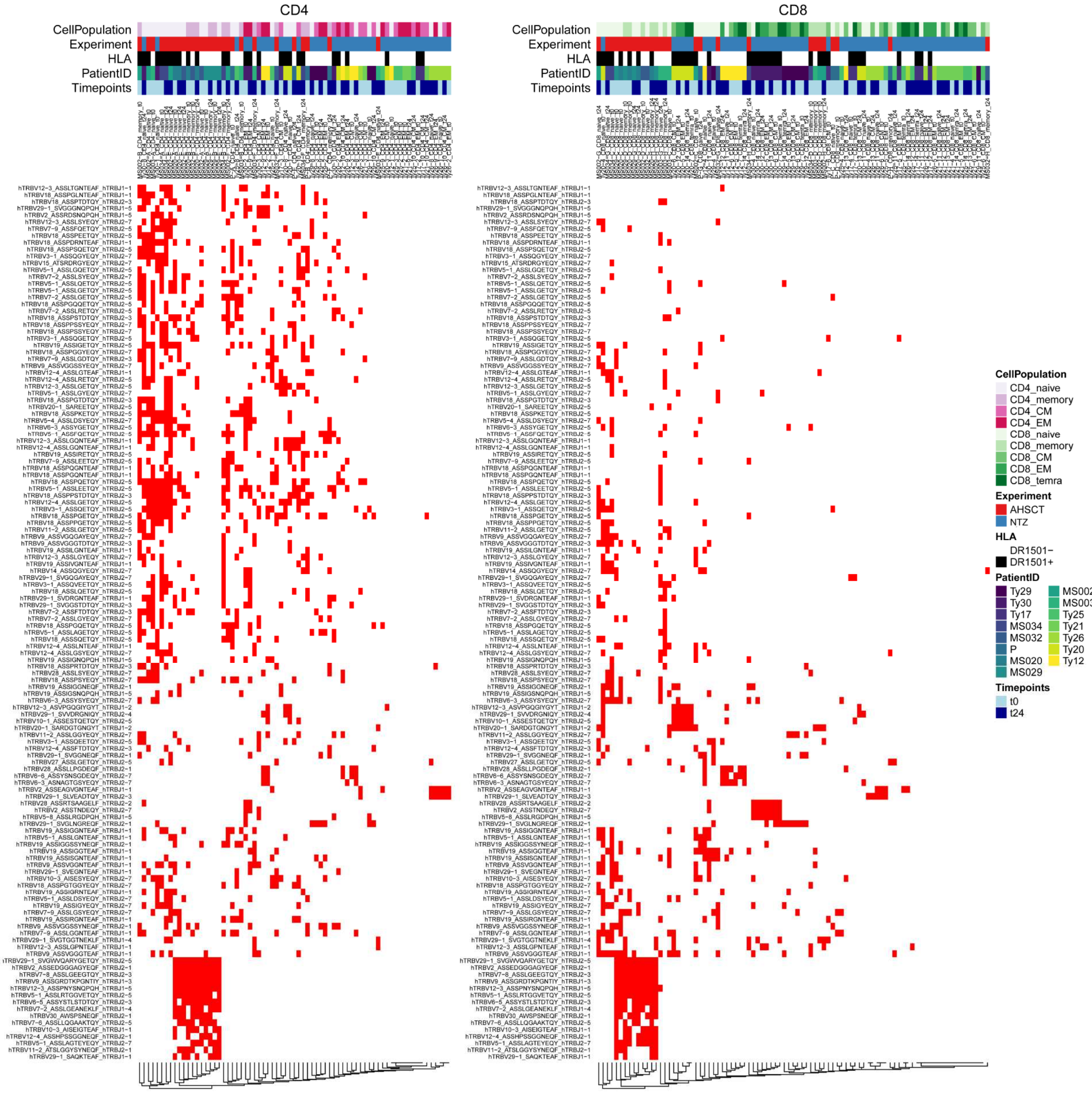

Figure S3. AHSCT repertoires have a higher number of highly shared public clones compared to natalizumab repertoires in CD4+ and CD8+ subpopulations. TCR clones shared among more than 10 samples visualized by treatment (AHSCT in red, NTZ in blue), by CD4+ (left heatmap; different shades of pink) or by CD8+ subpopulation (right heatmap; different shades of green), by patient ID, timepoint (t0-t24 in light and dark blue, respectively) and by HLA class II of patients when DR1501+ (white) or DR1501- (black). TCR clones are reported on the left and sample name on the top of the heatmaps.

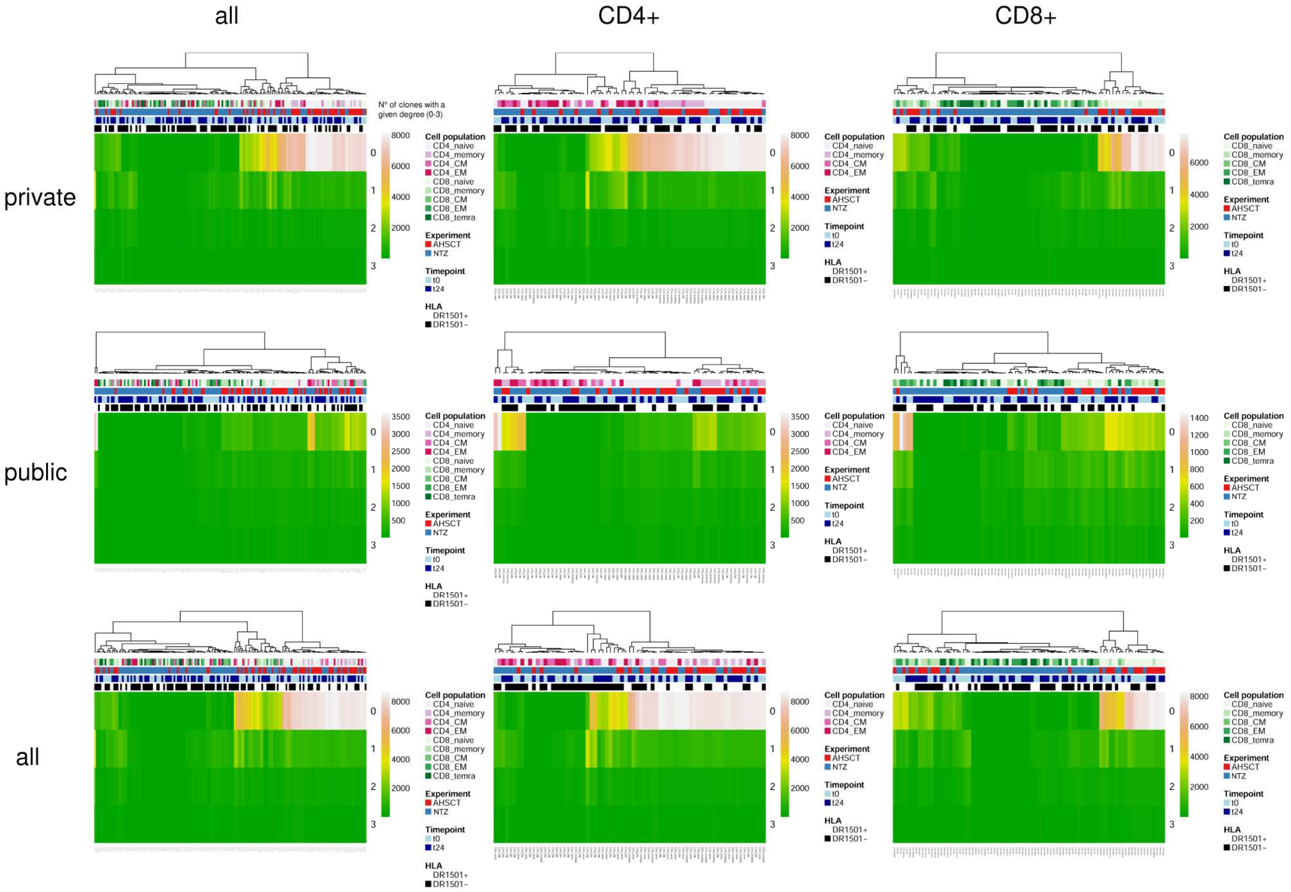

Figure S4. Natalizumab repertoires are characterized by a higher number of similar clones compared to AHSCT. Heatmaps of the degree distribution of private (first row), public (second row) and all clones (third row) of all T-cell subpopulations (first column), only CD4+ (second column) or only CD8+ (third column) subpopulation. The degree of a clone is the number of clones that is similar to (Levenshtein distance [LD] of 1: 1 amino acid [a.a.] change apart). Color bars indicate treatment (AHSCT in red, NTZ in blue), T-cell subpopulation (different shades of pink or green are reported for CD4+ or CD8+ subpopulations, respectively), timepoint (t0-t24 in light and dark blue, respectively) and HLA class II of patients when DR1501+ (white) or DR1501- (black). The heatmap x-axis labels indicate T-cell subpopulation and the y-axis labels indicate clones degree from 0 (no clones similar to a clone) to 3 (3 clones similar to a given clone). Hierarchical clustering was based on Euclidean distance.

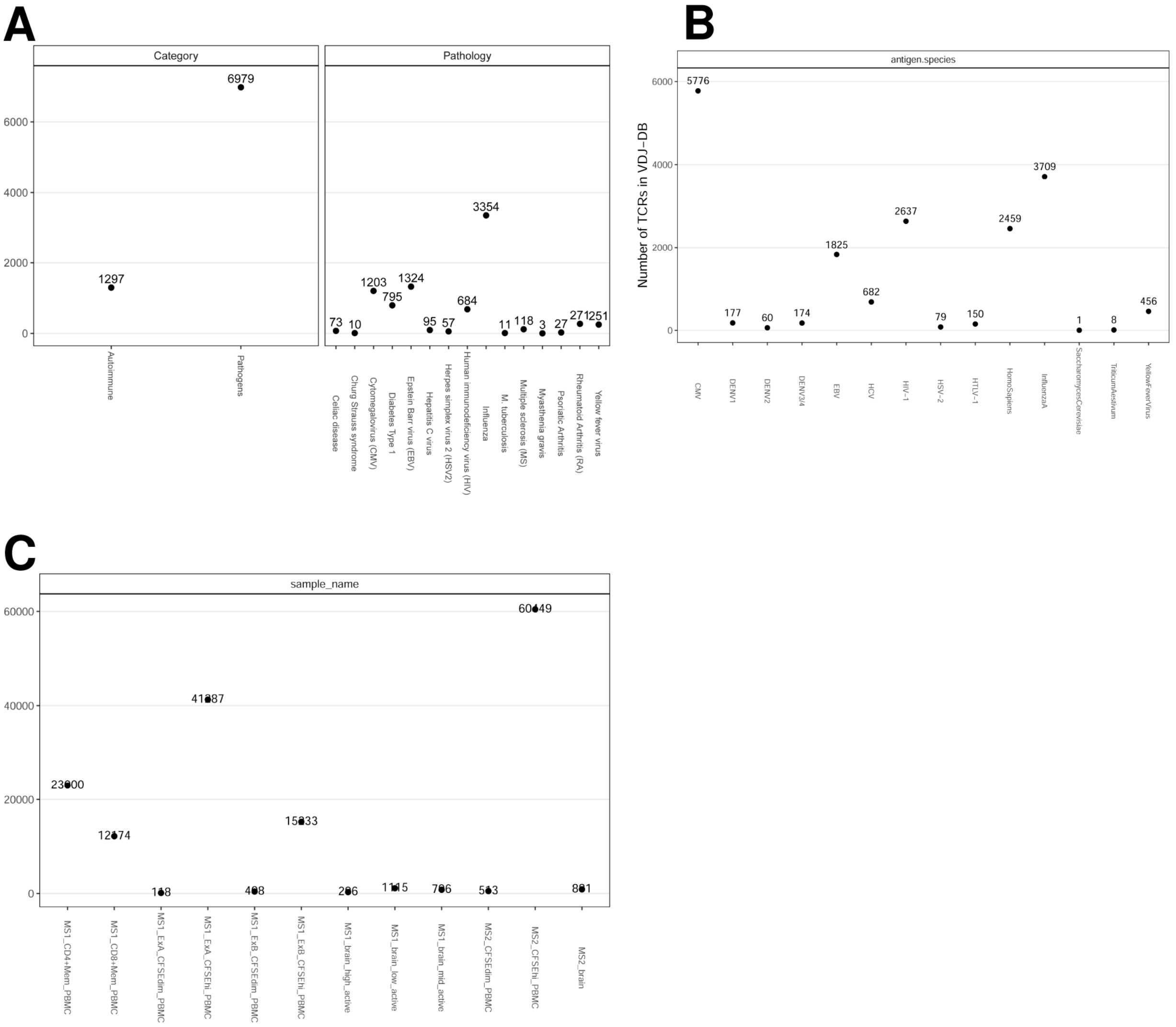

Figure S5. Characterization of McPAS-TCR, VDJdb and Jelcic et al. datasets. Absolute number of TCR sequences (TCRs) in McPAS-TCR (A), VDJdb (B) and TCRβ repertoires of two MS patients from Jelcic et al. (C). A) TCRs number of McPas-TCR database is reported by disease category, including autoimmune and pathogens (left panel) or by specific pathology (right panel). B) TCRs number of VDJdb is reported by antigen species. C) Number of TCRs found in Jelcic et al. MS dataset is reported by sample name (4). CFSE: Carboxyfluorescein Diacetate Succinimidyl Ester.

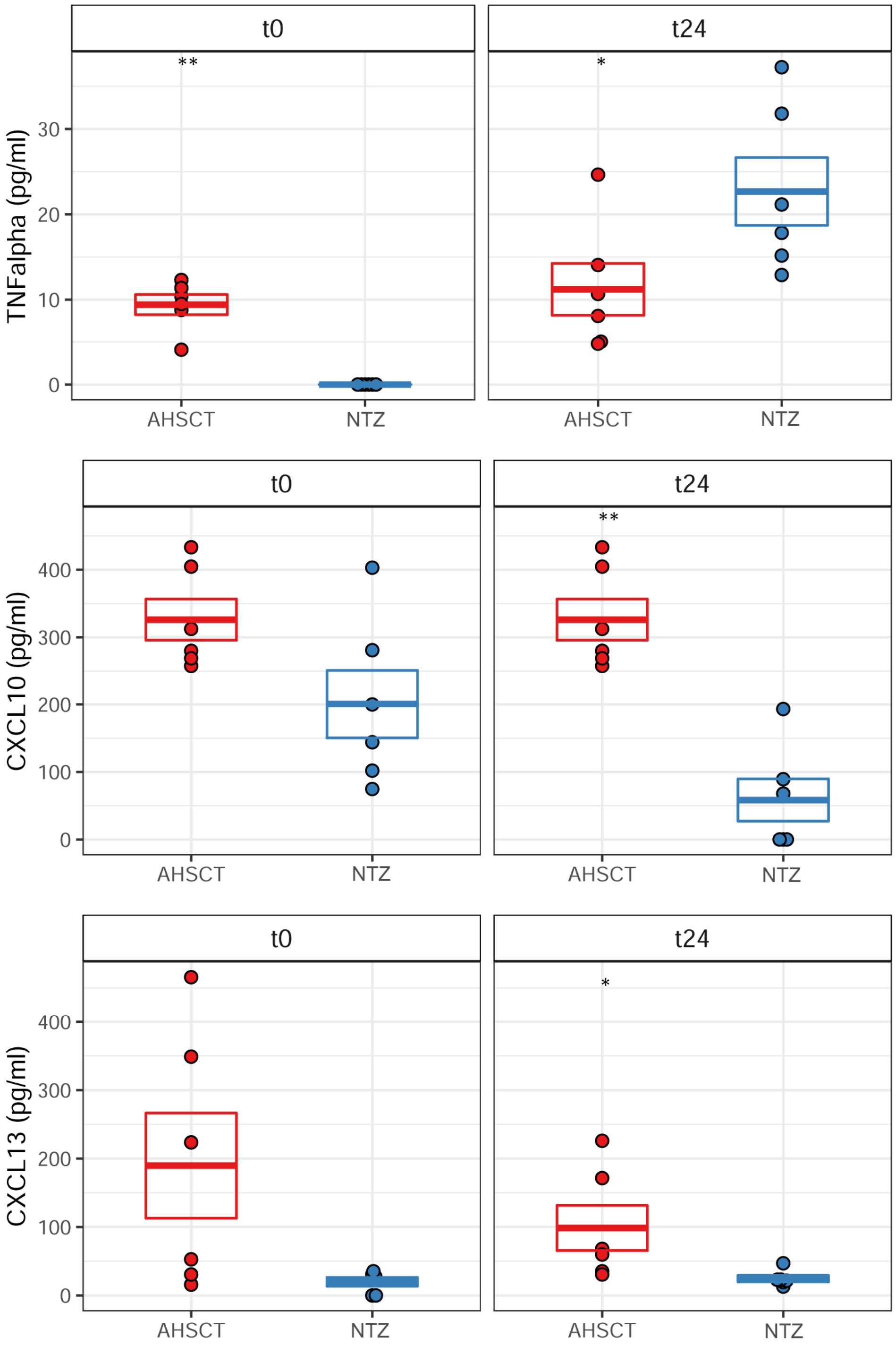

Figure S6. Serum cytokine levels differ between AHSCT and natalizumab patients. Panel reports, from the top to the bottom, TNFα, CXCL10 and CXCL13 levels (pg/ml) in serum samples of AHSCT (in red) and NTZ (in blue) patients at t0 (left graphs) and at t24 (right graphs). Each dot is a patient. Mean±SEM is reported. Statistical significance was determined by Wilcoxon test (*p<0.05; **p<0.01).
